# How tissue injury alarms the immune system and causes a systemic inflammatory response syndrome

**DOI:** 10.1186/2110-5820-2-27

**Published:** 2012-07-12

**Authors:** Jérôme Pugin

**Affiliations:** 1 Intensive Care - SIRS Unit, University Hospitals of Geneva, 1211, Geneva 14, Switzerland

**Keywords:** SIRS, Inflammation, Alarmins, Danger signals, Mitochondria, Pro-inflammatory cytokines

## Abstract

Systemic inflammation is very prevalent among critically ill patients, particularly those with extensive tissue injury. Although downstream mediators (cytokines) and effector cells (phagocytes) have been identified, proximal mediators originating from injured tissues remained elusive. Alarmins (“danger signals”) released by necrotic/injured cells have been identified recently and certainly play a role in triggering local and systemic inflammation in critically ill patients. The most promising alarmin candidates are of mitochondrial origin, i.e. mitochondrial DNA and the chemotactic factor fMet-Leu-Phe (fMLP). ATP also is released from necrotic tissues and stimulates the assembly of the inflammasome, leading to the production of proinflammatory cytokines, such as interleukin (IL)-1ß. The identification of novel alarmins opens new therapeutic avenues for the treatment of severe SIRS, and SIRS-dependent organ dysfunction.

## Review

### Introduction

Clinicians have recognized for a long time that critically ill patients often present with a stress response associating tachycardia and tachypnea, together with an “inflammatory” response characterized by fever and leukocytosis. The systemic inflammatory response syndrome (SIRS) concept was coined in 1991 by the late Roger C. Bone [1]. SIRS criteria were rapidly incorporated into the definition of sepsis during the first ACCP/SCCM consensus conference published two decades ago [2]. Sepsis was defined as bacterial infection with a systemic response, associating at least two of four SIRS criteria [3]. These definitions, although not perfect, still hold today, and are used in all sepsis studies, as well as at the bedside. Aside from its use in the sepsis definition, SIRS was rapidly recognized as a very prevalent syndrome in intensive care units (ICU), even in non-infected patients. Up to 93% of surgical ICU patients were reported to meet SIRS criteria, with ICU mortality ranging from 7–9% in the absence of an infection [3]. It also was epidemiologically demonstrated that SIRS may precede sepsis [4]. Because of its high prevalence in ICUs and the absence of a clear demonstration of a link between clinical SIRS and systemic inflammation, some of our colleagues have proposed to abandon the SIRS concept [5]. Interestingly, new tentative sepsis definitions also included a “response” item, like in the PIRO concept (predisposition, infection, *response*, and organ dysfunction) [6]. “Symptoms” such as heart rate, respiratory frequency, and band forms were retained in a PIRO score utile for stratification of critically ill patients and outcome prediction [7].

It was convincingly shown since the late 1980s that SIRS in critically ill patients was associated with the circulation of pro- and anti-inflammatory cytokines, even in the absence of infection [8]. It also was demonstrated that the balance of pro/anti-inflammatory mediators was pro-inflammatory in the inflamed organ (bioactive TNF and IL-1ß in lungs from ARDS patients, in ascitic fluid during pancreatitis, etc.) but was found to be anti-inflammatory in the circulation [9,10]. It was proposed that the normal response to local infection or tissue injury was to produce a *local* inflammatory reaction, attracting effectors phagocytes at the site of injuries or infection [11]. In contrast, an excess of anti-inflammatory mediators (soluble TNF receptors, IL-1RA, IL-10) prevented “systemic inflammation” within the circulatory compartment [11].

Although effector cells and their mediators have been described during SIRS (phagocytes, cytokines, chemokines) [12], what lacked in the paradigm of tissue injury-induced inflammation was the identification of endogenous mediators originating from injured tissues that were capable of generating this inflammatory/immune reaction [13]. It is the purpose of this review to discuss recent findings in the field of the pathogenesis of SIRS and the relevance of novel candidate endogenous danger signals.

#### The danger theory

The long time prevalent theory of the immune system was based on tolerance of self-antigens developed mostly during the fetal life by the deletion of auto-reactive lymphocytes in the thymus and bone marrow. In this theory, only remain during *extra utero* life immune cells capable of reacting to non–self antigens preventing autoimmunity, and conferring immunity to microbes and allografts. Sir Frank Macfarlane Burnet and Peter Medawar were awarded the Nobel Prize in 1960 for this innovative concept for self-/non–self- discrimination. Ample experimental evidence supported this paradigm; however, it appeared that it was somewhat reductive and could incompletely explain various situations of tolerance to foreign antigens or immune/inflammatory reactions to endogenous antigens. How to explain with the self-/non–self discrimination theory the absence of sepsis after tooth brushing where bacteremia is frequent? Why pregnant women do not reject their fetus, although half of its antigens coming from the father should be recognized as non-self? Why lactating women do not reject their breasts despite the expression in adulthood of “novel” milk proteins? In addition, because the immune system should be tolerant to self-antigens, how is autoimmunity explained?

Early in the 1990s, Polly Matzinger came up with the concept that the immune system did not care about self-vs. non-self but discriminated antigens based on the fact that were sensed as dangerous or not [14]. This “danger theory” may explain why pregnant women do not reject their nondangerous fetus and lactating women do not recognize newly expressed milk protein as dangerous. This also explains why circulating indolent gingival bacteria do not generate a sepsis syndrome after tooth brushing, because they are not seen as danger by our immune system. In this theory, what is essential for the immune system to react to an antigen is a signal of tissue injury, sensing the danger [15,16]. For the immune system to react, two signals are needed: one from the antigen and another one from tissue suffering. Vaccination is another example validating the danger model. Vaccines are usually efficacious only with a certain degree of tissue injury induced by the irritating effect at the site of injection of the adjuvant. The sole injection of the microbial antigen is usually well tolerated and does not induce a protective immune response. The danger theory was well captured by Polly Matzinger in an interview given to the New York Times in 1998: *“Imagine a community in which the police accept anyone they met during elementary school and kill any new migrant. That's the Self/Nonself Model. In the Danger Model, tourists and immigrants are accepted, until they start breaking windows. Only then, do the police move to eliminate them. In fact, it doesn't matter if the window breaker is a foreigner or a member of the community.”*[17]*.*

This danger theory also makes a lot of sense to the intensivist for several reasons [13]. SIRS could arise from an overwhelmed and systemic inflammatory reaction to massive release of danger signals from injured tissues. Examples of such situations in the intensive care unit are numerous: multiple trauma, pancreatitis, ischemia-reperfusion injury, shock, massive transfusions, and major surgery [13]. The devastating effects of sepsis may be explained by a synergistic activation of the inflammatory/immune system by foreign bacterial antigens and bacteria-induced tissue injury, further increased by shock, leading to organ dysfunction [13]. This synergism also may explain why patients with SIRS have such overwhelmed responses to bacterial infections acquired during their ICU stay.

#### Endogenous danger signals (alarmins)

What was lacking to support the danger theory was the identification of molecules originating from host cells signaling tissue injury to the immune system (alarmins, danger-associated molecular patterns, DAMPs). The definition of an alarmin could be a molecule released by injured cells/tissues that is capable of producing a local proinflammatory reaction, and attract to the site of injury effector cells such as phagocytes [18]. Alarmins not only may generate tissue inflammation after injury, but they also could act synergistically with microbial non–self-antigens to enhance the inflammatory reaction [19]. In case of massive tissue injury, the local production of alarmins could spillover into the circulation and produce systemic symptoms, such as fever, leukocytosis, as well as a stress humoral and neural response [19]. Leukocytes activated by alarmins may then relay and amplify the inflammatory process through the secretion of cytokines, the release of molecules, such as enzymes, lipid mediators, reactive oxygen, and nitrogen species [20]. The importance of tissue injury and the presence of microbial co-factors may account for the magnitude of the local vs. systemic inflammatory response (SIRS), vasodilation, and end-organ dysfunction.

Alarmins represent nothing less that the missing molecular link between tissue injury and the inflammatory response to tissue suffering. Conceptually, alarmins should be molecules present inside of cells sheltered from the immune system, and only released during tissue injury, most probably during cells necrosis or plasma membrane rupture [21]. Indeed, it has been shown that cell death by apoptosis does not produce inflammation. In addition, receptors for these danger signals should exist in leukocytes, and possibly in other cell types sensing the presence of endogenous alarmins that have become extracellular.

#### High mobility group box 1 (HMGB1)

Since the appearance of the concept of endogenous danger signals, several candidate alarmins have been proposed [18]. HMGB1 is a DNA chaperone concentrated in the cell nucleus first demonstrated to induce cell migration and differentiation. Its role in the pathogenesis of sepsis was reported in 1999 by the group of Kevin Tracey [22] and by others in the context of severe SIRS [23,24]. These investigators showed that HMGB1 was released by macrophages stimulated by lipopolysaccharide (LPS), tumor necrosis factor-α (TNF- α), and IL-1ß [22] and that high HMGB1 was measured in plasma from patients with septic shock [25]. Administration of antibodies to HMGB1 prevented late death in a murine model of sepsis [22]. In contrast, the injection of recombinant HMGB1 increased septic mouse lethality [22]. Since this pioneer work, many groups have shown a proinflammatory activity of HMGB1 and have identified putative surface leukocyte receptors for HMGB1 (RAGE, TLR2, and TLR4) [26,27]. It has been recently well established that most of–if not all–the inflammatory activity was not contained in the HMGB1 protein, but rather in bacterial contaminants bound to it, explaining TLR-dependent cell activation, HMGB1 only playing a role of chaperone for bacterial products, including LPS [28,29]. HMGB1 also can be released from dying cells and become a potential extracellular danger signal, but its role as an alarmin remains to be clearly demonstrated [30-32].

#### Heat shock proteins

HSPs are ubiquitous proteins, present in the cytoplasm, mitochondria, the cell nucleus, and act as molecular chaperones for proteins. They can be secreted by stressed cells and are released by necrotic cells [33]. The potential pro-inflammatory and immune-inducing effects of extracellular HSPs were first proposed in 1993, later thought to be mediated by TLR2 and TLR4 receptors. As with HMGB1, highly purified HSPs lacked cytokine function [29]. It is now believed that HSPs, like HMGB1, act more as molecular chaperones for bacterial products (LPS, flagellin, and lipopeptides) and co-factors for cell activation to pathogen-associated molecular patterns. Therefore, the role of HSPs and HMGB1 in sterile inflammation and SIRS remains elusive [28,29].

#### Other putative alarmins

S100 proteins (also called calgranulins) are a group of calcium-binding proteins that have been proposed as potential alarmins, particularly those expressed in phagocytes (S100A8, -A9, and -A12) through their interaction with RAGE and TLR4 [34,35]. Again, no evidence exists to show a clear danger signal effect of these proteins in the absence of bacterial products. Crystals of uric acid were shown to induce the assembly of the NALP3 inflammasome, leading to the cleavage of pro-IL-1ß into mature IL-1ß by caspase-1 [36]. Whereas uric acid is now recognized as a local danger signal in the pathogenesis of gout arthritis, none reported a role of this alarmin in systemic inflammation. Recently, another alarmin released by necrotic cells has been proposed: IL-33 [37]. This mediator acts through the receptor ST2 at the surface of CD8^+^ T cells, enhancing T cell clonal expansion and was shown to be necessary for the control of RNA and DNA viral infections in mice [37].

#### Mitochondrial alarmins

**Figure 1:**
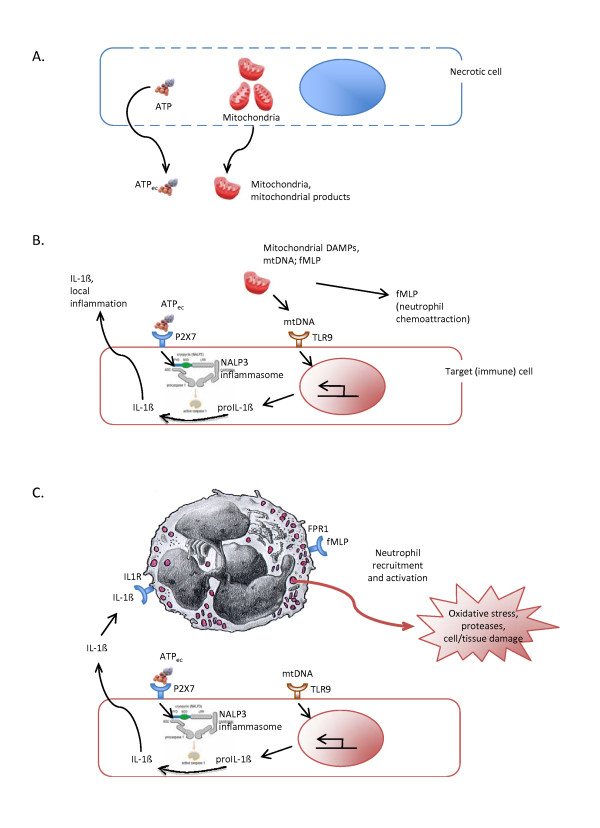
**Inflammation induced by alarmins released from injured/necrotic cells.A.** Injured/necrotic cells with membrane breaks release ATP and mitochondrial alarmins (mitochondrial products). **B.** Extracellular ATP stimulates in target (immune) cells the assembly of the NALP3 inflammasome via its interaction with the P_2_X_7_ receptor, activating caspase-1. Mitochondrial (mt)DNA stimulates the transcription of the pro-IL-1ß gene via its interaction with TLR9 receptor, and the production of pro-IL-1ß that will be cleaved by activated caspase-1 into bioactive IL-1ß, which in turn can be released into the alveolar space and the interstitium and generate local inflammation. fMLP will create chemotactic gradients to attract neutrophils to the injured tissues. **C.** Neutrophils attracted to the injured tissues will be activated further by fMLP and IL-1ß via their respective receptors (FPR1, and IL-1R), generate oxidative burst and release proteases, which could be further deleterious to tissues (neutrophil-dependent tissue injury).

Mitochondria are ancient bacteria that have become endosymbionts and then organelles specialized with the evolution into oxygen-dependent energy producing factories (ATP production, cellular respiration) [38]. Two important features of mitochondria are that: 1) they have retained some molecules of bacterial origin throughout the evolution, and 2) they escape the immune system due to their obligatory intracellular location but can potentially be recognized as non-self when they are found extracellularly. Evidence has recently accumulated that mitochondrial alarmins mediate inflammation [39]. It was recently demonstrated that mitochondrial DNA (resembling to bacterial DNA) was found extracellularly after tissue injury, in the plasma from multiple trauma patients [40]. MtDNA, like bacterial DNA, is proinflammatory via the activation of Toll-like receptor 9 present in many cell types. The formulated peptide fMet-Leu-Phe (or fMLP) is another molecule present in the wall of both bacteria and mitochondria, but absent in any other human structure. It was shown that the release of mitochondria during tissue injury attracted phagocytes via the chemoattractant fMLP, through its ligation to the fMLP receptor (FPR1) present at the surface of neutrophils [40]. By far, mitochondrial DAMPs are to date the best candidate endogenous danger signals; they respond to all alarmin criteria [18,41]. Injected to animals, mitochondrial preparations induce lung injury resembling to that seen during ARDS [40]. A schematic view of the action of mitochondrial danger signals acting as alarmins is proposed in Figure 1.

#### Extracellular ATP

Tissue injury/necrosis also releases cytoplasmic adenosine triphosphate (ATP), which becomes “extracellular” ATP (ATP_ec_), and activates neighboring cells via the leukocyte P_2_X_7_ receptor (Figure 1) [42]. Like uric acid, ATP_ec_ activation of cells leads to caspase-1 activation and maturation of IL-1ß via the assembly of the NALP3 inflammasome and participate in local inflammation [42]. Assembling the NALP3 inflammasome probably does not suffice to create inflammation, because the caspase 1 substrates pro-IL-1ß and pro-IL-18 need to be present to generate inflammation [43]. ATP_ec_ might therefore be seen more as a co-alarmin, enhancing the inflammation induced by mitochondrial alarmins or bacterial products such as lipopolysaccharide responsible for the transcriptional activation of the pro-IL-1ß and pro-IL-18 genes [43].

## Conclusions

The discovery of potent endogenous danger signals released from injured/necrotic cells clearly adds to our understanding of the pathogenesis of “sterile inflammation,” as well as SIRS when tissue injury is sufficient to produce systemic inflammation. Alarmins represent nothing less than the proximal endogenous mediators linking cell necrosis and the inflammatory response generated by tissue injury. The identification of alarmins from mitochondria that are ancient bacteria sheltered inside of cells makes a lot of sense. Mitochondria have retained throughout the evolution some molecules of their bacterial ascent that, when found extracellularly, can be recognized as “non-self” by cells from the immune system. Other alarmins, such as ATP, also are strictly intracellular molecules that when found extracellularly will be sensed as a “danger molecule,” meaning for the immune system: “rupture of plasma cell membrane and tissue injury.” Although extracellular ATP does not seem to be capable of mediating a great deal of inflammation, it is an important cofactor, an enhancer of the inflammatory reaction in response to mitochondrial danger molecules and to microbial-associated molecular patterns.

Given their proximal location in the inflammatory cascade during tissue inflammation, alarmins and alarmin-dependent pathways represent attractive targets to develop drugs aimed at dampening deleterious inflammatory reactions following (massive) tissue injury. Examples of such situations in critical care are: multiple trauma, ARDS, ventilator-induced lung injury, ischemia/reperfusion injury, and severe pancreatitis. Blocking alarmin-dependent pathways will certainly dampen neutrophil-dependent tissue injury. It also will decrease innate immune responses and possibly favor bacterial and yeast superinfections, because it will interfere with the recruitment and the activation of neutrophils. Phagocyte recruitment at the site of tissue injury also plays an important role for the removal of cell debris, the restitution of tissue integrity and healing. As with many modulators of the inflammation, finding the right dose of “anti-alarmins,” the proper route of administration, and the adequate time window will be key to develop successful novel drugs designed to combat SIRS and SIRS-induced organ dysfunction.

## Abbreviations

ATP_ec_: Extracellular adenosine triphosphate; fMLP: Formyl-Met-Leu-Phe; PIRO: Predisposition, infection, response, and organ dysfunction; IL-1RA: IL-1 receptor antagonist; DAMPs: Danger-associated molecular patterns; HMGB1: High mobility group box 1; RAGE: Receptor for advanced glycation end products; TLR: Toll-like receptor; HSPs: Heat shock proteins; LPS: Lipopolysaccharide; MtDNA = Mitochondrial DNA; NALP3: NACHT, LRR and PYD domains-containing protein 3.

## Competing interests

The author declares that he has no competing interests.
